# A Defective Viral Particle Approach to COVID-19

**DOI:** 10.3390/cells11020302

**Published:** 2022-01-17

**Authors:** Maria Kalamvoki, Vic Norris

**Affiliations:** 1 Department of Microbiology, Molecular Genetics and Immunology, University of Kansas Medical Center, 3901 Rainbow Blvd, Kansas City, KS 66160, USA; 2 Laboratory of Microbiology Signals and Microenvironment, University of Rouen, 76821 Mont Saint Aignan, France; victor.norris@univ-rouen.fr

**Keywords:** antivirus, immunity, therapy, coronavirus, defective interfering particle, aptamer, COVID-19, extracellular vesicle, synthetic defective viral genome

## Abstract

The novel coronavirus SARS-CoV-2 has caused a pandemic resulting in millions of deaths worldwide. While multiple vaccines have been developed, insufficient vaccination combined with adaptive mutations create uncertainty for the future. Here, we discuss novel strategies to control COVID-19 relying on Defective Interfering Particles (DIPs) and related particles that arise naturally during an infection. Our intention is to encourage and to provide the basis for the implementation of such strategies by multi-disciplinary teams. We therefore provide an overview of SARS-CoV-2 for a multi-disciplinary readership that is specifically tailored to these strategies, we identify potential targets based on the current knowledge of the properties and functions of coronaviruses, and we propose specific strategies to engineer DIPs and other interfering or therapeutic nanoparticles.

## 1. Introduction

In a Holliday lecture in 1999, Don Ganem explained that pandemic infection is a recurrent, not a temporary, phenomenon: “The supervention of the AIDS pandemic put the lie to all of these optimistic predictions about how infectious disease was conquered and was no longer a problem. Now we know, of course, that notion was foolish to begin with, that infectious disease, epidemic infection, is a part of the human condition. I’m going to show you that it’s really a part of human evolution that we can never get away from infectious disease as a class. We can triumph over individual infectious diseases, but the concept that we’re going to be free of infection as a species is a ridiculous one and one that nobody believes anymore” [1]. The current COVID-19 pandemic is a reminder that the need to develop new weapons to combat infections is as pressing now as then.

A powerful, anti-viral strategy could exploit the DIPs and related particles that arise naturally for every family virus. DIPs are particles containing degenerate forms of the virus genome, which interfere with the replication of the parental virus but are non-replicative *per se*. Historically, such particles were considered artefacts of virus propagation in vitro; however, studies have shown that defective viral genomes are present in patients infected with viruses such as hepatitis C, influenza A and respiratory syncytia [2,3,4]. Thus, DIPs are currently investigated for their potential role in influencing disease outcomes and shaping virus evolution. A similar form of interference is observed when small viruses such as satellite viruses and virophages parasitize the larger viruses with which they are associated thereby decreasing their fitness. Other particles that can be produced during an infection include the virus-like particles (VLPs). These particles are composed of viral structural proteins, they morphologically resemble the parental virus but are non-infectious due to lack of genetic material. Another type is the extracellular vesicle (EV). EVs are released by all types of cells as they facilitate intercellular communication. During an infection they communicate virus-specific signals.

DIPs reduce virulence, induce high levels of interferons, and promote viral persistence by mechanisms that are not well understood [5]. In the case of RNA viruses, the shorter genome of the particle may allow it to out-compete the wild-type virus [6]. Alternatively, specific changes to the particle’s genome confer advantages over the full-length genome in using a limiting viral or host factor [7]. Also, the particle’s genome may interfere with the assembly of the wild-type’s genome into virions [8]. Furthermore, the particles may stimulate the host immune system [9]. Finally, the particles may compete with the wild-type virus for entry or cause internalization of the virus entry receptor inhibiting virus entry and spread [5]. 

Therapeutic interfering particles (TIPs) are a class of DIPs engineered to reduce the severity of viral diseases [10]. TIPs could be designed to use the same transmission routes as the wild-type virus thereby limiting viral transmission in populations at risk. Given that DIPs are diverse, it is unclear which design of TIP would prove most effective. Single-cell studies have revealed the importance of phenotypic diversity in a large number of systems. It is likely that this also applies to viral infections; hence, the diverse population of DIPs actually benefits both the virus and host. A novel anti-viral strategy might take this into account by constructing a diverse population of synthetic defective viral genomes (synDVGs) with prophylactic or therapeutic potential. In addition, the properties of EVs and VLPs could be harnessed to suppress SARS-CoV-2 infection and mitigate pathogenesis. These particles could be engineered to trigger antiviral responses, alleviate inflammation, enable tissue regeneration, or interfere with macromolecular assemblies during SARS-CoV-2 infection.

Finally, it is likely that the design, construction, implementation and analysis of strategies based on therapeutic nanoparticles will entail multi-disciplinary collaborations. In what follows, we therefore provide a non-comprehensive overview of SARS-CoV-2 (Overview Section) that is related to these strategies. We discuss potential targets of the different types of nanoparticles described above and describe strategies to engineer nanoparticles with different properties. We collectively term the different types of nanoparticles “therapeutic nanoparticles” because the overarching goals are to suppress infection and alleviate disease.

## 2. SARS-CoV-2 DIPs, VLPs and Other Nanoparticles

Defective Viral Genomes (DVGs) can be formed during the replication of a virus when the polymerase switches between different templates or skips parts of the same template. Discontinuous transcription of CoV genomes enables recombination in a cell coinfected with more than one CoV species or variants via strand switching by the viral RdRp [11,12,13,14,15,16,17]. Some RNAs produced following such recombination procedures gather characteristics of DVGs including deletions that range from <1 kb to >20 kb; these DVGs retain intact 5′- and 3′-UTRs, and can be amplified by the CoV replication transcription complex (RTC) provided in trans by a helper virus. These recombination events have been attributed to the 3′–5′ exoribonuclease activity of the proofreading nsp14 protein and are responsible for CoV evolution. Packaging of DVGs into viral particles results in DIP production.

Another particle produced during CoV infections is the VLP. VLPs are self-assembled nanostructures composed of the structural proteins of a virus that, due to a lack of genetic material, are non-infectious. The data regarding the minimum requirements for the formation of SARS-CoV-2 VLPs and the ability of the membrane protein (M) alone to be secreted are controversial. However, co-expression of M with either the nucleocapsid (N) or the envelope (E) protein appears to be sufficient for VLP formation, while M, N and E together are required for optimal VLP production [18,19]. VLPs could advance our understanding of the assembly requirements for SARS-CoV-2, but could also be used for vaccine or interference strategies [20,21,22,23,24,25].

Finally, an analysis of plasma-derived nanoparticles from COVID-19 patients demonstrated that they were enriched in pro-inflammatory cytokines, IFN-γ, peptidases and proteases involved in vascular remodelling, and markers of cardiovascular tissue injury [26]. These nanoparticles could augment pro-inflammatory responses, endothelial dysfunction and thrombosis, which have been observed in severe COVID-19 cases.

## 3. Strategies

### 3.1. Targeting Macromolecular Assemblies

It has been persuasively argued that many cellular functions are performed by high-level complexes, modules or “supermolecules” [27,28]. Hence, those assemblies associated with viral processes can be targeted. The replication of coronaviruses involves the assembly of membrane-bound replicative organelles in which two lipid bilayers are closely paired [29]. This pairing is induced by the ribonucleoprotein complex proteins, which have been localized to networks of convoluted membranes and vesicles [30]. Targeting this network is important because viral polymerases that have a decreased fidelity often have an increased production of defective viral genomes [31,32,33]. Markers such as the Green Fluorescent Protein, chromobodies, or the haemagglutinin tag could be used to localize DVGs with the ability to encode proteins [34].

Poisoning complexes—and thereby disrupting functions—could be achieved in several ways. One way is via the production of novel or truncated peptides. Such production occurs in deletion DVGs derived from influenza [35]. DVGs constructed with mutations in the conserved regions, by which the influenza A polymerase subunits interact, inhibited polymerization and reduced virulence [36]. The multimerization of the polymerase is essential in the production of DVGs during influenza virus infection [32]. Such multimers might be perturbed either by altering the stoichiometry of coronavirus proteins or by generating incomplete RNAs or incomplete proteins that interfere with associations between macromolecules. For example, in the case of stoichiometry, this depends in part on post-translational regulation such as the phosphorylation of the serine and arginine residues in the SR-region of the SARS-CoV-2 N protein; a synDVG could therefore be created with this region deleted. In the case of incomplete proteins, deletion of the C-terminus of the N protein might disrupt the oligomerization of both the mutant and the wild-type protein. Sequestration of key constituents could be achieved by constructing synDVGs containing multiple copies of regulatory sequences, as illustrated by the sequestration of components of the Tat-based transcriptional activation system of HIV-1, where a vector was used that contained multiple copies of the sequences to which Tat binds [37]. Given the potential value of a recombinant protein, it may be worth considering the construction of a TIP to encode fusion of the ACE2 fragment to the M-protein to perturb SARS-CoV-2 assembly.

### 3.2. Targeting RNA

The transcription-regulating sequences (TRSs) at the 3′ end of the leader sequence (TRS-L) that precedes each viral gene (TRS-B) contains a conserved core sequence (CS) of 6–7 nucleotides along with variable 5′ and 3′ flanking sequences; as the conserved core sequence is identical for the genome leader (CS-L) and all mRNA coding sequences (CS-B), the CS-L may form a base-pair with the nascent negative strand complementary to each CS-B during the template-switching, which is central to transcription and replication [38]. The TRSs are therefore good candidates for targeting both viral transcription and replication.

### 3.3. Deletions

synDVGs constitute a powerful approach to viral therapy. Those that have deletions of part of the wt-genome could outcompete the wt-virus for the proteins and lipids essential to infectivity, which means wild-type virus release would be lower. Also, the synDVG could be released and transmitted to other cells where it could again hinder the replication of the wild-type virus. The factors to consider when designing synDVGs include the complex relationship between the length of the DVG, the degree of interference with the wild-type virus and the number of effective DVGs released. The minimum for a deletion DVG would be to have the 5′-UTR and 3′-UTR and the secondary structures they adopt to allow replication by the RdRp. Depending on whether the objective is to maximize production of DVGs or maximize interference, there is likely to be more than one optimum size. For example, the tight regulation of the quantity of each sub-genomic mRNA, which is believed to be important for the correct stoichiometry of the proteins of the wild-type virus, could be perturbed by a synDVG with partial deletions so that it encodes some proteins but not others. 

The nature of the deletions in naturally occurring DIPs is useful in the design of synDVGs. An analysis of DVGs isolated from a respiratory syncytial virus indicated that the generation of copy-back mutations was not completely random but resulted from specific sequences encoded in the viral genome [39]. The analysis of the 5′ and 3′ regions flanking deletion sites during influenza infection was also consistent with conservation of specific sequences and structures [3,40]. 

A synthetic defective interfering SARS-CoV-2 was developed using the 5′ UTR and the adjacent 5′ part of nsp1 in ORF1a, the nsp15 that includes the putative packaging signal and the sequence spanning the 3′ part of the N sequence, ORF10 and the 3′-UTR. The rationale of this design is that a long ORF enables defective interfering genomes in some CoVs to replicate more efficiently and, since multiple transcriptional regulatory sequences (TRS) reduce replication efficiency, the 3′ portion was chosen to start within the N sequence to exclude its TRS. This synthetic defective genome was found to replicate three times faster than SARS-CoV-2 thereby reducing the viral load. Moreover, it transmitted as efficiently as the full-length genome, confirming the putative packaging signal of SARS-CoV-2 [41]. Based on this principle, two TIPs were developed recently that could inhibit SARS-CoV-2 in primary human lung organoids and in the Syrian Golden Hamster model of SARS-CoV-2 following intranasal delivery. These TIPs also reduced pro-inflammatory cytokines, and prevented pulmonary edema. The mechanism of SARS-CoV-2 inhibition by these TIPs was proposed to be due to competition for viral trans elements and no stimulation of innate immunity was recorded [42]. 

The use of a cocktail of different synDVGs directed against one or different targets may be more effective than a single synDVG. This is not only because synDVGs may act synergistically but also because the probability of the virus escaping inhibition by mutating is reduced. These “cocktail” synDVGs would be constructed so that they could neither complement one another nor recombine to generate the wild-type virus. In the case of HIV, antiviral genes were constructed that contained Tat- and Rev-binding decoys that acted synergistically [43], while a therapeutic vaccine, DermaVir, has been designed to boost T cell responses specific to 15 HIV antigens expressed from a single plasmid DNA [44,45]. 

Cocktails of synDVGs could have another advantage. There is no reason to suppose that viruses might cause only one pandemic at a time. We should therefore anticipate that two or more different viruses will eventually cause concurrent pandemics. Such a scenario could be dealt with by using a cocktail containing synDVGs to several different viruses. The success of such treatment would not depend on prior knowledge of the virus infecting or risking infecting a particular individual.

### 3.4. Immune Stimulation by TIPs

In designing an anti-viral therapy, one approach would be to construct a synDVG based on SARS-CoV-2 containing sequences resembling the copy-back sequences that stimulate the immune system. Alternatively, heterologous copy-back DVGs able to stimulate innate anti-viral immune responses strongly could be used. In the case of the Sendai virus (SeV), DVGs with copy-back genomes appear to be better at stimulating the immune system than those with a deleted genome [46]; although this was attributed to their long stretches of dsRNA, it has been argued that other characteristics of copy-back DVGs are also important contributors to the induction of anti-viral responses as shown by the induction of type 1 IFNs by the 44 nucleotide (nt)-long stem-loop motif in the copy-back genome of the DVG-546 Sendai virus [47]. SeV-based, copy-back DVGs increase the antigen presentation capacity of mouse and human dendritic cells, which increases the activation of T cells whilst, in the case of influenza A and respiratory syncytial virus, experimental vaccines with an adjuvant containing SeV-based, DVGs delivered subcutaneously, intramuscularly or intranasally have an increased level of antibodies and anti-viral protection [5,48,49,50]. 

A general purpose DVG might be developed based on the DIP 244 which was derived naturally from genome segment 1 of influenza A; this not only inhibits influenza viruses via RNA interference but also has a broad-spectrum activity against all other interferon-sensitive respiratory viruses via stimulation of type I interferon and pro-inflammatory cytokines [9,51,52,53]. It should be noted that SARS-CoV-2 is particularly sensitive to recombinant human IFN-α and IFN-β, which reduce viral titers [54]. Indeed, co-infections with IAV DIPs and SARS-CoV-2 led to abrogation of SARS-CoV-2 replication in a JAK/STAT-dependent mechanism [55]. Similarly, EVs from HSV-1 were found to restrict the respiratory syncytia virus (RSV). Thus, defective virus particles or other nanoparticles released from virus-infected cells could restrict heterologous viruses most likely through innate immunity activation [56].

### 3.5. Anti-Sense Oligonucleotides, Aptamers, Ribozymes, and Antibodies

Anti-sense RNA, RNA aptamers and ribozymes could all be incorporated into a TIP as could the RNA coding for the epitope-binding part of an antibody.

#### 3.5.1. Anti-Sense Oligonucleotides

The anti-sense oligonucleotides (ASOs) strategy involves the use of nucleic acid strands of approximately 20 nt that can specifically hybridize to the complementary sequence of the target RNA [57,58]. The fate of the ASO:RNA hybrid varies depending on the ASO design strategy and either it could lead to cleavage of the mRNA, alter splicing, or it could form a steric blockade resulting in disruption of translation. Morpholino-type ASOs targeting the TRS in the 5′-UTR of the SARS-CoV block virus replication [59]. ASOs targeting conserved regions of the CoV genome such as the RdRp or the N sequence can be used to bypass issues of increased mutagenesis. An ASO-based strategy could be used to improve the effectiveness of treatments based on nucleoside analogues by splicing out ExoN [60].

Delivery of ASOs has been achieved using cationic polymers or by modifying them with lipids so they assemble in nanomicelles [61,62,63]. Phages are ideal vehicles for transferring nucleic acids, because they have the advantages of simple production, purification and a large capacity for containing genetic material. VLPs from bacteriophage Qβ have been used to encapsidate target RNAs to detect viral infections, including foot-and-mouth disease virus (FMDV) or Ebola virus. Asuragen and SeraCare have announced developments of SARS-CoV-2 positive controls for diagnostics, in which a SARS-CoV-2 detection module for RT-PCR was encapsidated into VLPs from the bacteriophage Qβ and the CCMV virus [64]. RNA nanoparticles of the bacteriophage φ29 have been used to deliver therapeutic oligonucleotides [65].

DNA rich in non-methylated CpG motifs are immunostimulatory and can be used as vaccine adjuvants or to stimulate protective immunity against pathogens. To enhance their stability and reduce serious side effects, a packaging and delivery strategy using VLPs has been proposed. These oligonucleotides induce protective cytotoxic T cell responses in the absence of systemic side-effects; hence, VLPs mounting protective immunity could accelerate SARS-CoV-2 clearance [66].

An alternative is to take a gene therapy approach, in which RNA oligos are encoded in a viral vector such as adeno-associated virus (AAV). Thus, short, hairpin RNAs can be fused to small PolIII promoters such as tRNA genes. The PolIII transcripts would be exported to the cytoplasm where the shRNAs would function in the RNAi pathway to knock down expression of various RNAs.

#### 3.5.2. Aptamers

Nucleic acid aptamers are artificial, single-stranded or double-stranded DNA or RNA that can bind to their targets [67,68,69]. Because of their binding specificity, aptamers are often compared to antibodies [70,71]. Those that bind to viral proteins can have diagnostic and therapeutic potential. For example, aptamers binding to haemagglutinin have been used to detect different influenza strains [72]. Single-stranded DNA aptamers that bind to the ZIKA NS1 protein have diagnostic potential [73]. DNA aptamers against the dengue virus envelope protein can neutralize infection by all four serotypes of the virus [74]. RNA and DNA aptamers that disrupt the interaction of HSV-1 glycoprotein D with the virus entry receptor interfere with virus entry into the cells [75,76]. RNA aptamers were also described against the HIV-1 Gag protein that perturbed the Gag-genomic RNA interaction leading to the inhibition of HIV-1 genomic RNA levels [77]. Aptamers can also be used to suppress the activity of viral enzymes or host targets that contribute to pathogenesis [78,79]. Two RNA aptamers specific to the polymerase of HCV inhibited the initiation and the elongation of viral RNA synthesis by competing for the binding sites of the polymerase with viral RNA template [80].

RNA aptamers of approximately 40 nt were described for the SARS-CoV NTPase/helicase. The aptamers could inhibit the dsDNA unwinding activity of the helicase but not the ATPase [78]. A ssDNA aptamer that binds the N protein of SARS-CoV was proposed for diagnostic purposes [81]. This aptamer can also bind the N protein of SARS-CoV-2 [82]. Considering that the N protein is critical for nucleocapsid assembly, to antagonize host antiviral responses and the RNAi machinery, the aptamer targeting N protein could be repurposed to interfere with N functions [83,84]. Indeed, the C-terminus of the N protein contains a highly positively charged region possessing a strong affinity for ssDNA, ssRNA and dsDNA that can be easily targeted by aptamers. Also, an aptamer that binds nucleolin was found to inhibit SARS-CoV-2 replication [85]. Nucleolin is hijacked by the virus for its replication and the aptamer inhibits this process. Aptamers that bind the S protein have been designed mostly for diagnosis [86,87]. Aptamers that bind to the receptor-binding domain of S that could potentially inhibit virus entry were recently reported [88]. Aptamers that target the different methyltransferases of the virus, endonucleases and proteases are predicted to be effective.

Aptamers can be linked to therapeutic oligonucleotides such as siRNAs, miRNAs, and gRNAs forming chimeras that improve the properties of the therapeutic oligonucleotides [89]. RNA aptamer siRNA chimeric molecules could be designed to target the RNA genome/transcript of SARS-CoV-2.

#### 3.5.3. Ribozymes and Antibodies

In the case of HIV, inactivation was achieved via anti-sense sequences against *rev* to prevent replication [90]: the Gag component of the capsid was fused to a calcium-sensitive nuclease to inactivate viral nucleic acids [91,92] and the 5′ leader was cleaved by a ribozyme [93]. These related technologies and DVGs based on SARS-CoV-2 could be used to target one or more of the RNA targets discussed above [94,95,96,97]. For example, the CRISPR/Cas13 RNA knockdown system delivered by the adeno-associated virus was used to cleave the SARS-CoV-2 RNA genome using guide RNAs to target the sequences encoding ORFab and the S-protein [98]. A single-domain camelid antibody against the S-protein can neutralize the SARS-CoV-2 pseudovirus [99]; such nanobodies could be encoded by a TIP.

### 3.6. EVs

EVs facilitate cell-to-cell communication and are produced by all types of cells. Recent studies demonstrated that COVID-19 patients had increased circulating platelet-derived EVs [100]. These EVs could transport platelet-derived cytokines and other proinflammatory molecules, including damage-associated molecular patterns [101]. The contribution of these EVs to COVID-19-associated coagulopathy and lung injury remains undetermined [102].

**Figure 1:**
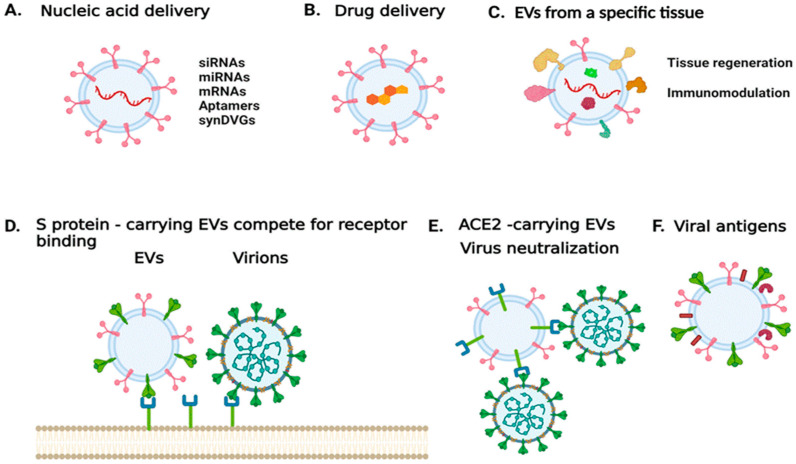
Harnessing EV properties to combat SARS-CoV-2 infection or treat COVID-19. (**A**) EVs can be used to deliver nucleic acid sequences that either modulate the expression of specific targets, express genes of interests, or encode for viral products. (**B**) EVs can be used to deliver compounds of interest. (**C**) EVs derived from a specific cell type or tissue could be used to mitigate disease and trigger tissue regeneration. (**D**) EVs carrying the spike protein can be used to antagonize viral entry into a host cell. (**E**) EVs carrying the virus entry receptor ACE2 could serve as decoys for the virus. (**F**) EVs carrying viral antigens could be used for vaccine development. The image was generated with BioRender.com (accessed on 13 September 2021).

While EVs produced during an infection can exacerbate pathogenesis, those produced by uninfected cells could be modified and used for therapeutic purposes (Figure 1). EVs can be engineered to serve as carriers of nucleic acid sequences, including siRNAs, aptamers, genomes of TIPs, or other inhibitory molecules including proteins and small molecule inhibitors [103,104]. EVs would be more attractive if they could be directed to the desired targets. EVs produced by a particular type of cell, such as an immune cell, could target the proteins on the surface of diseased cells or an inflammatory immune cells [105]. Other strategies to redirect EVs include the expression of antibodies on the surface of EVs that target surface molecules in recipient cells or the expression of ligands to specific receptors.

In the case of SARS-CoV-2, vesicles could be designed to carry the S protein to antagonize virus entry [106]. Alternatively, EVs released during SARS-CoV-2 infection were found to carry ACE2; such EVs could be used to treat infections by coronaviruses that rely on ACE2 binding to enter host cells [107]. In addition, mesenchymal/stromal stem cell (MSC)-derived EVs have been shown to naturally target injured tissue and ameliorate acute organ injury [108]. These MSC EVs have been suggested for promoting recovery in patients with ARDS [109]. Thus, MSC EVs that carry ACE2 receptors could have a decoy function for SARS-CoV-2, while mitigating ARDS [110]. Currently, there are four clinical trials exploring the use of MSCEVs [111]. One trial aims to determine the therapeutic potential of aerosol inhalation of adipose-tissue derived MSC EVs in patients with severe COVID-19. The safety profile of this treatment is assessed in a different trial. An additional trial aims to determine whether MSC EVs can suppress immune system over-response to the virus, and whether they can trigger regenerative processes. In other trials, bone marrow-derived MSC EVs are being tested in COVID-19 patients with moderate-to-severe ARDS [111].

Finally, viral antigens on EVs could also be used for vaccine development or to serve as adjuvants. Depending on the status and the type of cell from which they originated, EVs may facilitate the initiation, expansion, maintenance, or silencing of adaptive immune responses [112].

### 3.7. Mimics

One possibility would be to produce the ACE2 receptor fragment, to which SARS-CoV-2 binds, on the surface of a bacteriophage or a bacterium to act as a competitor; these phages or bacteria might then be used to impregnate masks and coat other surfaces, including skin and mucosal membranes, or even be used as inhalants. There is a novel technique that could be used to produce peptides that would bind proteins on the surface of the virus and, indeed, that would bind the other viral proteins (and that could then be encoded by a synDVG). This technique is the *Mimic Chain Reaction* [113] which, in a sense, is the peptide equivalent of the PCR technique. The *Mimic Chain Reaction* is based on the auto-induction or quorum-sensing systems of bacteria and allows both the selection of peptides that bind to a target and peptides that mimic the epitopes of the target.

## 4. Problems

### 4.1. Constructing Large synDVGs

Synthesizing long sequences of nucleic acids has long been considered difficult. Error rates during DNA synthesis are typically 0.5% per nucleotide and particular difficulties arise when the sequence requires multiple, consecutive, repetitions of the same nucleotide or a stretch rich in C and G. 

Now, however, it is relatively straightforward to synthesize sequences of 30 kb. The phosphoramidite-based technology employed by Twist Bioscience (San Francisco, CA, USA) allows the simultaneous synthesis of up to 10,000 different DNA fragments each 200 nucleotides long, which can then be joined. Agilent’s machines are capable of synthesizing 244,000 DNA fragments simultaneously and those of Thermo Fisher, 35,000. Several companies (Nuclera Nucleics, Ansa Biotech, Spindle Biotech, Molecular Assemblies, Merck, and DNA Script) are developing a technology based on Terminal Deoxynucleotidyl Transferase. Helixworks is now believed capable of synthesizing DNA sequences longer than 2000 nucleotides in one go. Catalog’s machine recently converted 16 gigabytes of the English text version of Wikipedia into DNA in about 12 h. Hence it should be possible to make the DNA sequences of many synDVGs end to end in a day. Of course, a DNA sequence corresponding to the synDVG genome still needs to be transcribed into RNA and contain all the required sequences for assembly into the virion itself.

### 4.2. Obtaining DIPs That Attenuate Rather Than Exacerbate Disease Progress

Despite the fact that many studies are focusing on the therapeutic potential of DIPs, examples demonstrating that DIPs can exacerbate symptoms have been reported. It was shown that the proportion of defective hepatitis B virus genomes was higher in patients with severe liver disease compared to those with milder disease. Perhaps the outcome of an infection in the presence of DIPs depends on a balance between the immunostimulatory potential of the DIPs versus the host response. Some DIPs may shift this balance by activating strong immune responses that could trigger a cytokine storm and augment disease progression. 

Another mechanism for disease exacerbation occurs when DIPs interfere with vaccines. DIPs and wild type virions have a high antigenic similarity, so DIPs compete with the virus to bind host-produced antibodies that would otherwise bind and neutralize the virus. The mechanisms implicated in disease severity by DIPs are multiple and remain poorly understood.

### 4.3. Avoiding Loss of EV Activity and Other Complications

The different EV purification methods and selected cargo packaging could result in the loss of EV activity [104]. EVs produced by infected cells could be a double-edged sword as interwoven relationships exist between the biogenesis of EVs and virion envelopment. Also, viruses often hijack or divert EV biogenesis pathways to communicate virus-specific signals to surrounding cells. Furthermore, EV population dynamics could shift an infection outcome, as the milieu of the infection is enriched in an EV population with proviral and antiviral roles in different ratios [56,114,115]. Another challenge is that during an infection, virus-like particles (VLPs) can be formed. In the case of infection by coronaviruses, these VLPs are composed of the virus’s structural proteins M, S, E, N but they lack the viral genome. Different types of VLPs lacking one, two, or three structural proteins have been reported. The potential role of these VLPs on virus infection and virus-mediated pathogenesis currently remains unknown. A final complication is that EVs carrying viral components may exacerbate pathogenesis. This is the case for EVs released from EBV-infected cells that carry the viral oncoprotein LMP-1, which contributes to EBV-associated malignancies [116]. Similarly, EVs released from Karposi’s sarcoma-associated herpesvirus (KSHV)-infected cells can cause cell proliferation, migration and transcriptome reprogramming of EV-recipient cells [117]. Despite the complications, EVs are evolving as a powerful tool.

### 4.4. Avoiding Loss of Aptamers

Major problems can arise when using aptamers. The first is rapid degradation, particularly of RNA aptamers. Nuclease-resistant aptamers can be developed by incorporating modified nucleotides and by developing “mirror aptamers” [118]; second is the rapid removal of the aptamer from the bloodstream by renal filtration. A solution to this problem is based on conjugating aptamers to polyethylene glycol (PEG); third is cross-reactivity of aptamers with molecules of similar structure. To avoid this, a negative selection step with structurally similar molecules can be performed; fourth is the delivery of aptamers to intracellular target molecules because most aptamers are selected for molecules on the cell surface or in the bloodstream. Delivery of aptamers to intracellular targets can be achieved using viral vectors or through receptor-mediated endocytosis. Strategies to overcome issues related to the duration and regulation of aptamer activity and automation in their production keep evolving.

## 5. Discussion

Herein we propose to harness properties of different types of nanoparticles that are naturally produced by SARS-CoV-2-infected and uninfected cells for SARS-CoV-2 inhibition and COVID-19 treatment. We collectively term these nanoparticles “therapeutic nanoparticles”. One approach relies on the use of DIPs and TIPs that compete with the wild-type virus for entry, replication, assembly, and egress. The same type of particles could be used to enhance antiviral responses for viral clearance. An alternative approach involves the use of engineered EVs that can carry the genome of TIPs, small interfering RNAs, viral entry receptor(s), viral antigens, immunostimulatory molecules and anti-inflammatory factors. VLPs offer another approach that could be used to antagonize viral entry into the cells or stimulate immune responses. 

The proposed approach underlying the development of therapeutic nanoparticles is based on population dynamics. This is because we first consider the problem as arising from the complex web or ecology of interactions among multiple players (e.g., various types of host cells, the heterogeneous population of viruses and naturally occurring DVGs), and second we advocate acting on this ecology by constructing a variety of therapeutic nanoparticles to target these players and their interactions in different ways. The design of therapeutic nanoparticles and synDVGs would benefit from a better understanding of the complex interplay between the host immune system and the heterogeneous population of viruses and DIPs. Indeed, it may turn out that DIPs are emerging naturally in the present pandemic and are responsible for attenuating symptoms in some individuals [119].

In the “population dynamics” approach to therapeutic nanoparticles and synDVGs, it is worth noting that the presence of DIPs in vaccines appears to increase both the efficiency of the vaccine and its safety [5]. It might therefore be expected that using a cocktail of nanoparticles and synDVGs in the presence or absence of a wild-type virus could both reduce its replication and spread and provide many epitopes for the immune system.

Finally, improving techniques, in the case of aptamers or nanobodies, or emerging techniques, in the case of the *Mimic Chain Reaction* [113], could be used to generate the sequences needed for synDVGs to inhibit a variety of viral targets.

## 6. SARS-CoV-2 Overview: Viral and Host Targets

### 6.1. Spike (S) and Viral Entry into the Host Cell

**Figure 2:**
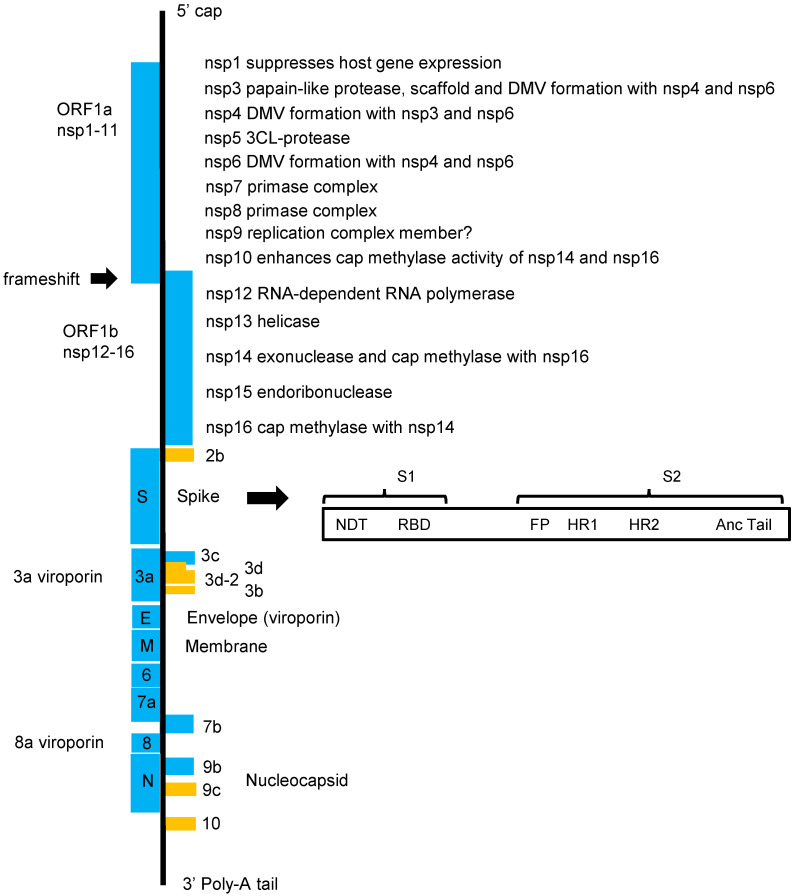
The SARS-CoV-2 genome. On entry into the host cell, a coronavirus particle is uncoated, and its single-stranded positive-sense RNA genome enters the cytoplasm. Two-thirds of the coronavirus genome is occupied by two large overlapping open reading frames (ORF1a and ORF1b) that are translated into polyproteins and that are processed to generate 16 non-structural proteins (nsp1 to nsp16). The rest of the genome includes ORFs for the structural proteins and several accessory proteins. The 5′-UTR is 265 nucleotides long, while the 3′-UTR is 358 nucleotides. The major distinction between other coronaviruses related to SARS-CoV and SARS-CoV-2 is in orf3b, Spike and orf8 but especially in the highly variable Spike S1 and orf8, which are recombination hot spots.

SARS-CoV-2 entry into the cells is a profound target as it involves macromolecular assemblies that include virus entry machinery and host receptor complexes, enzymatic processes that alter the conformation of the virus entry machinery from a pro-fusogenic to a post-fusogenic state, and other biochemical processes that lead to the fusion of the viral membrane with the cellular [120,121,122]. The spike (S) viral glycoprotein mediates entry to the cells. It is produced as a precursor that trimerizes and is cleaved by furin-like proteases into the receptor-binding fragment (S1) and the fusion fragment (S2). S1 binds to the viral entry receptor. A second cleavage occurs in the S2 by the transmembrane serine protease 2 (TMPRSS2), or by the endosomal cathepsins B and L that causes dissociation of the receptor-binding fragment and the irreversible refolding of the fusion fragment into a stable post-fusion conformation. The post-fusion conformation of the cleaved fragment (S2′) is a trimeric hairpin structure, containing the heptad repeat 1 (HR1) and heptad repeat 2 (HR2) regions, which form a six-helical bundle (Figure 2) [120,121,122]. The HR region is a potential antiviral target as it is conserved among HCoVs and is essential for fusion events. A synthetic lipopeptide that binds to the HR1 region appears to inhibit infection by different CoVs [123]. Generally, peptides derived from the HR2 region of class I viral fusion proteins of enveloped viruses appear to bind competitively to viral HR1 and inhibit infection [124]. 

### 6.2. Nucleocapsid (N)

The N protein plays a central role in transcription, replication and encapsidation of the viral RNA [125]. It consists of three highly conserved domains: the N-terminal domain (NTD) which associates with the viral genome; the C-terminal domain (CTD) involved in RNA binding and protein oligomerization; an intrinsically disordered central serine/arginine (SR)-rich linker that is heavily phosphorylated [30,126]. The N protein forms dimers that are arranged into octamers via the CTD and can further assemble into larger intertwined filaments. The ribonucleoprotein complexes are incorporated into the forming viral particles via interactions with the M protein [127]. The N protein is also involved in interferon inhibition, actin reorganization, cell cycle progression and apoptosis [83]. Thus, potential targets of the N protein include the NTD to inhibit RNA-binding [128], the self-binding domain of N to inhibit oligomerization, the kinase and the SR-rich region to inhibit synthesis of the full-length genome, and the binding site on the N for the M protein to inhibit virion assembly.

### 6.3. Envelope (E)

E is an integral membrane protein present at a low amount in the virion that is important for particle assembly; this role is attributed to its induction of membrane curvature and its interaction with the M protein [129,130]. Indeed, the E protein and the M protein together are sufficient for production of VLPs [131,132]. The E protein assembles as a pentameric viroporin-like protein that functions as an ion channel [133]. It also contains a PDZ-binding motif (PBM) allowing it to bind cellular proteins [134]. Disruption of the homotypic interactions that lead to channel formation, the interactions through the PBM motif, and the binding of E to M will inhibit infection. Small molecules interfering with the ion channel activity will negatively impact infection and perhaps mitigate pathogenesis [135].

### 6.4. Membrane (M)

M is the most abundant protein in the CoV particle, and it is essential for virion formation. It is the primary driver of virus budding as it oligomerizes and forms a lattice structure at the ERGIC membranes. M protein alone, or together with either E or N, appears to form VLPs [18,19]. During virion formation, the S and E proteins are integrated into the lattice through lateral interactions with M, whereas the N protein and the RNA interact with the cytoplasmic domain of M [136]. Thus, antagonizing the interactions between M and the other virion components will negatively affect SARS-CoV-2 infection.

### 6.5. RNA-Dependent RNA Polymerase (RdRp) (nsp12) and the Co-Factors nsp7 and nsp8

RdRp is responsible for the replication and transcription of the RNA genome, and it helps viruses to escape host defences by acquiring mutations. The activity of RdRp is enhanced by accessory proteins. Nsp7 and nsp8 form the primase complex, which activates and enhances the primer-dependent activity of RdRp and also increases RdRp template binding [137]. Nsp14 provides the exoribonuclease activity for proofreading that the RdRp lacks. In replicating the viral genome, access of RdRp is facilitated by sequences and structures at the 3′ end of the RNA. Replication of positive-sense genomic RNA requires RNA elements in both the 5′ and 3′ ends of the viral genome [38]. Thus, potential targets to suppress SARS-CoV-2 infection include domains involved in the interaction with accessory proteins, template RNA and rNTP binding sites, and the catalytic active site. TIPs and other nanoparticles could be used to deliver different types of interfering molecules.

### 6.6. Non-Structural Protein 1

SARS-CoV-2 nsp1 acts as a translation inhibitor by binding 40S and 80S ribosomes and reducing the pool that is available for translation. Under such ribosome-limiting conditions, mRNAs with a more efficient 5′-UTR gain an advantage [138,139].

The SARS-CoV-1 nsp1 appears to employ an additional mechanism to suppress host gene expression that involves host mRNA degradation, but this mechanism has not been confirmed yet for the SARS-CoV-2 nsp1. Among the transcripts found to be destabilized following SARS-CoV-1 nsp1 expression are those encoding type I interferon components. 

Thus, antagonists targeting the Nsp1-40S ribosome interaction are expected to decrease SARS-CoV-2 replication and render the virus vulnerable to immune clearance [139]. 

### 6.7. Non-Structural Protein 9

Nsp9 is essential for SARS-CoV replication. Nsp9 has RNA- and DNA-binding ability and most likely is a member of the viral replication complex and perhaps regulates viral RNA replication and transcription. Nsp9 dimerizes via a conserved α-helical ‘GXXXG’ motif. Substitutions within this motif reduce RNA binding and SARS-CoV replication [140,141,142]. Thus, the residues involved in RNA binding can be targeted.

### 6.8. Viroporins

SARS-CoV-2 encodes three viroporins: E (see above), ORF3a and ORF8a.

#### 6.8.1. Open Reading Frame 3a 

ORF3a contributes to pathogenesis through increased virulence, infectivity, and virus release [134]. ORF3a is a transmembrane protein that localizes at the Golgi but can also traffic to the plasma membrane. ORF3a forms an ion channel that is linked to its proapoptotic activity, NLRP3 inflammasome activation, pro-inflammatory responses and antagonizes type I IFN responses [143,144]. Dominant negative mutants that sequester host interactors of ORF3a or that interfere with the channel formation will obstruct ORF3a function. 

#### 6.8.2. Open Reading Frame 8

ORF8 is an accessory protein that has less than a 20% sequence similarity with SARS-CoV ORF8a/b [145]. ORF8 is a highly immunogenic, immunoglobulin-like protein that can suppress type I interferon responses, inhibit the presentation of viral antigens by MHC-I, and take part in pulmonary inflammation and fibrogenesis [146]. The crystal structure of SARS-CoV-2 ORF8 indicates that the protein can form large-scale assemblies, but their biological significance remains unclear. 

### 6.9. Viral Proteases

#### 6.9.1. Papain-like Protease (PLP2 or nsp3)

Nsp3 has multiple domains. First, the ubiquitin-like domain 1 (Ubl1) binds ssRNA containing AUA patterns [147], and its deletion abrogates CoV replication. Ubl1 (and Ubl2) may interact with Ub or ISG15 by mimicking their shape. Ubiquitination and ISGylation are involved in anti-viral responses and protein degradation and nsp3 through mimicry could interfere with ubiquitinated or ISGylated host targets, leading to disruption of host-antiviral signals.

Second, a Glu-rich domain may be involved in metal-ion binding, DNA/RNA mimicry and protein–protein interactions, Third, an X domain acts as a hydrolase by removing mono- and poly(ADP-ribose) from modified proteins, also termed “de-MARylation” and “de-PARylation”, respectively. ADP-ribosylation is involved in various cellular processes, including innate immunity activation [148]

Fourth, is the ubiquitin-like domain 2 (Ubl2), a second ubiquitin-like subdomain. Fifth, is the papain-like protease, which is responsible for releasing nsp1, nsp2, and nsp3 from the N-terminal region of polyproteins 1a and 1a/b. It also exhibits de-ubiquitinase activity [149,150] and cleaves the IRF3 blunting type I interferon responses. Sixth, are the transmembrane regions (TM1 and TM2) that along with nsp4 and nsp6 drive the formation of replication organelles. Nsp3 also serves as a scaffold for the assembly of the membrane-associated replication/transcription complex (RTC). Thus, nsp3 is an attractive target for inhibiting SARS-CoV-2 infection since peptides that antagonize the function of individual domains, small molecule inhibitors or dominant negative forms of the protein-disrupting macromolecular assemblies could have therapeutic potential [151,152].

#### 6.9.2. Main Protease (M^pro^) or 3C-like Protease (3CL^pro^) or nsp5

Nsp5 is indispensable for viral replication as it mediates processing of viral polyproteins in at least 11 conserved cleavage sites, including its own proteolysis. Nsp5 is composed of three domains: domains I and II resemble the architecture of chymotrypsin and the substrate binding site is located in the cleft between these two domains; domain III mediates nsp5 dimerization. Nsp5 also cleaves host substrates including mediators of innate immunity and inflammation. Cleavage of these substrates is speculated to contribute to the production of IL-6 and inflammatory responses observed during COVID-19. The crystal structure of nsp5 from different coronaviruses indicates a high degree of conservation of the substrate-binding site, which makes it an attractive antiviral target [153,154,155].

### 6.10. Viral Endonucleases, Helicase and S-Adenosylmethionine (SAM)-Dependent Methyltransferases (MTases)

#### 6.10.1. Non-Structural Protein 14

Nsp14 carries a 3′–5′ exonuclease (ExoN) and a guanine-N7 methyl transferase (N7-MTase) [156]. The ExoN activity corrects errors made by the RdRp. ExoN-deficient mutants display a hyper-mutation phenotype with decreased sub-genomic RNA populations and increased defective viral genomes (DVGs). ExoN is important in viral RNA synthesis and viral fitness [57,157]. 

Nsp14 contains a conserved motif (S-adenosyl-L-methionine-binding motif or SAM) that is important for introducing the 5′-cap of the viral RNA. Mutations within the SAM motif that abrogate the N7-methyltransferase activity without affecting the ExoN activity have detrimental effects on the infection as the stability of the viral mRNA decreases [158]. The N7–MTase activity of nsp14 is enhanced following the nsp10 binding. 

The ExoN activity has been implicated in viral resistance to the nucleoside analog remdesivir and Ribavirin [154,159,160,161,162]. Impairment of the ExoN activity will result in a higher mutagenesis rate during virus replication and will render the virus more susceptible to nucleoside analogs. Also, inhibition of the N7-methyltransferase activity will reduce the translation efficiency of the viral mRNA.

#### 6.10.2. Non-Structural Protein 16 and nsp10

For SARS-CoV-2 RNA cap formation, the nsp10, nsp13, nsp14 and nsp16 proteins are involved. Nsp13 is the helicase that unwinds the viral RNA during replication and also possesses a 5′-RNA triphosphatase activity that cleaves the 5′end of the nascent RNA to provide a diphosphate. Nsp14 and nsp16 are responsible for cap methylation. Activities of both MTases is enhanced by nsp10. Nsp10 stabilizes the SAM-binding pocket of nsp16 and extends its substrate RNA binding groove. An nsp10 peptide derived from its region that binds nsp16 could suppress nsp16 activity and replication of SARS-CoV [163,164]. The 2-O’-MTase activity of nsp16 is indispensable for replication of CoVs and therefore is considered an attractive target.

2′-O-methylation of RNA cap structures is a mechanism that pathogens have evolved to circumvent interferon-stimulated genes. Loss-of-function mutations in the catalytic site of nsp16 resulted in decreased viral replication and increased sensitivity to type I interferons. Such attenuated mutant viruses have potential as vaccines as they retain immunogenicity and confer protection in vivo against challenge with pathogenic coronaviruses [165].

Inhibition of the 2′-O-MTase activity of nsp16 by either preventing binding of its co-factor nsp10 or by loss-of-function mutations can affect the viral mRNA by depriving it of a cap structure. These effects will decrease mRNA stability and reduce its translation efficiency.

#### 6.10.3. Non-Structural Protein 13

Nsp13 has a helicase activity that catalyzes the unwinding of double-stranded oligonucleotides using energy from hydrolysis of NTPs. Nsp13 unwinds both RNA and DNA duplexes in the 5′ to 3′ direction [166]. All natural nucleotides and deoxynucleotides can be used as substrates by the cognate NTPase activity of the helicase [167,168]. Nsp13 is essential for the replication of CoVs and its sequence is conserved [169]. The helicase and ATPase activities of nsp13 increase after binding to the replication-transcription complex (nsp12, nsp8 and nsp7). Thus, approaches to inhibit nsp13 include inhibition of the ATP-binding domain, of the NTPase activity, of nucleic acid binding to the helicase domains, of helicase translocation and also of the interaction between nsp13 and nsp12 [166].

#### 6.10.4. Non-Structural Protein 15

Nsp15 is an endoribonuclease (EndoU) that facilitates the evasion of viral double-stranded RNA recognition by RNA sensors. EndoU cleaves the 5′-polyU from negative-sense viral RNA, termed PUN RNA. The protein cleaves dsRNA substrates on the 3′ end of U in unpaired regions. PUN RNA activates MDA5-dependent interferon responses leading to a suppression of the infection. The crystal structure of nsp15 demonstrated that it forms a hexamer. In addition, the endonuclease active site is structurally similar to RNase A, and small molecule inhibitors of RNase A inhibit nsp15. Mutations in nsp15 affected viral replication leading to greatly attenuated disease in mice. Thus, expression of dominant negative mutants with impaired EndoU activity are expected to antagonize the ability of SARS-CoV-2 to suppress RNA sensors [170,171,172].

### 6.11. Viral Replication Organelles

#### 6.11.1. Non-Structural Proteins nsp4 and nsp6

The membrane-spanning proteins nsp3, nsp4 and nsp6 divert production of host endomembranes into double-membrane vesicles (DMVs), which are the replication organelles [152,173]. Nsp6 of SARS-CoV and MHV facilitates generation of autophagosomes from the ER but limits their expansion at the stage of omegasome formation [174,175]. Similar to these coronaviruses, control of autophagy by SARS-CoV-2 nsp6 could turn out to be a mechanism of the virus to evade adaptive immunity and other host responses [176].

Nsp4 interacts with nsp3 and possibly host proteins to initiate the rearrangement of ER membranes and induce membrane curvature to form DMVs. This interaction is essential for viral replication. Thus, disruption of the nsp3/nsp4 interaction and expression of nsp6 dominant negative mutants that cannot hijack autophagy pathways are expected to disrupt replication.

#### 6.11.2. Lipid Composition of Viral Replication Organelles

Lipids are fundamental to the life cycle of viruses as they are involved in viral entry into the cells [177], in the assembly and functioning of the viral replication complex [178,179], in fuelling viral replication [180], and in determining the distribution of viral proteins [181]. Coronavirus replication entails the formation of double-membrane vesicles and other membranous structures that provide a scaffold for the viral replication/transcription complexes and that sequester these complexes away from antiviral host factors [182]. An idea of the lipid requirements of coronaviruses comes from the finding that, in cells infected with the HCoV-229E, levels of lysophosphatidylethanolamine and arachidonic acid were raised with the linoleic acid-to-arachidonic acid pathway being perturbed [183]. In addition, the lipid synthesis pathways regulated by the sterol regulatory element binding protein are required for the palmitoylation of viral proteins and the formation of double-membrane vesicles [184].
